# Reviewer acknowledgement 2013

**DOI:** 10.1186/1480-9222-16-2

**Published:** 2014-01-20

**Authors:** Daniel Shanahan

**Affiliations:** 1 BioMed Central, 236 Gray's Inn Road, London WC1x 8HB, UK

## Abstract

**Contributing reviewers:**

A peer-reviewed journal would not survive without the generous time and insightful comments of the reviewers, whose efforts often go unrecognized. *Biological Procedures Online* has been blessed by the support of highly-qualified peer reviewers, and the Editor-in-Chief, Shulin Li, and staff of the journal would like to show their appreciation by thanking the following for their invaluable assistance with review of manuscripts for the journal in Volume 15 (2013).

## 

Maqusood Ahamed

Saudi Arabia

Joel Bumgardner

United States of America

Wim DHaeze

United States of America

Marxa Figueiredo

United States of America

Michael George

United States of America

Yan Guo

United States of America

Ronald Jackson

United Kingdom

Shisheng Li

United States of America

Bolin Liu

United States of America

QI Liu

United States of America

Mauro Provinciali

Italy

Tetsuo Shibuya

Japan

Debbal Sidi Mohammed

Algeria

Todd Stedeford

United States of America

Hong-Bo Xie

Australia

Jianhua Yang

United States of America

Shaomian Yao

United States of America

Liangfang Zhang

United States of America

